# Early PET‐CT in patients with pathological stage III colon cancer may improve their outcome: Results from a large retrospective study

**DOI:** 10.1002/cam4.1818

**Published:** 2018-10-22

**Authors:** Assaf Moore, Olga Ulitsky, Irit Ben‐Aharon, Gali Perl, Yulia Kundel, Michal Sarfaty, Ron Lewin, Liran Domachevsky, Hanna Bernstine, David Groshar, Nir Wasserberg, Hanoch Kashtan, Noa Gordon, Aaron Sulkes, Baruch Brenner

**Affiliations:** ^1^ Institute of Oncology, Davidoff Cancer Center Rabin Medical Center Petach Tiqva Israel; ^2^ Sackler Faculty of Medicine Tel Aviv University Tel Aviv Israel; ^3^ Department of Nuclear Medicine Rabin Medical Center Petach Tiqva Israel; ^4^ Department of Surgery B Rabin Medical Center Petach Tiqva Israel

## Abstract

**Background:**

Current staging of pathological stage III colon cancer (CC) is suboptimal; many patients recur despite unremarkable preoperative staging. We previously reported that early postoperative PET‐CT can alter the stage and management of up to 15% of patients with high‐risk stage III CC. This study aimed to determine the role of the test in the general stage III CC population.

**Methods:**

A retrospective study of all consecutive patients with stage III CC who underwent early postoperative PET‐CT between 2005 and 2017.

**Results:**

A total of 342 patients, 166 (48.5%) males, median age 66 years (range, 29‐90), were included. Pathological stage was IIIA, IIIB, and IIIC in 18 (5.3%), 257 (75.1%), and 67 (19.6%) patients, respectively. Median number of positive lymph nodes was 2 (range, 0‐32). PET‐CT results modified the management of 46 patients (13.4%): 37 (10.8%) with overt metastatic disease and 9 (2.6%) with a second primary. The 5‐year disease‐free survival for true stage III patients was 81%. The median overall survival for the entire cohort and for true stage III patients was not reached and was 57.2 months for true stage IV. Of the 37 patients found to be metastatic, 14 (37.8%) underwent curative treatments and 9/14 (64.3%) remain disease‐free, with a median follow‐up of 83.8 months. Predictive factors for upstaging following PET‐CT were identified.

**Conclusion:**

Early postoperative PET‐CT changed the staging and treatment of 13.4% of stage III CC patients and has the potential for early detection of curable metastatic disease. Outcome results are encouraging. Prospective validation is ongoing.

## 1 INTRODUCTION

Stage III colon cancer (CC), that is, CC with regional lymph node involvement, is a common and potentially lethal global health problem. Its standard therapy involves complete resection of the primary tumor and draining lymph nodes and postoperative adjuvant chemotherapy.^1^, ^2^ In spite of the significant progress in the treatment of stage III CC, many of these potentially curable patients will still develop metastatic disease even after unremarkable preoperative staging, standard surgery and optimal postoperative adjuvant therapy, and succumb to their disease.^2^ Clearly, better staging and treatment methods are needed for this common medical problem.

The use of ^18^Fluorodeoxyglucose (FDG) Positron Emission Tomography‐Computed Tomography (PET‐CT) has allowed a more accurate staging and improved the management of various cancers and multiple clinical settings. However, its use in CC is currently limited to evaluation of unclear findings in conventional imaging during preoperative staging or follow‐up, work‐up for rising carcinoembryonic antigen (CEA) levels and preoperative staging of potentially resectable liver metastases.^1^, ^3^, ^4^ According to current guidelines, the routine use of PET or PET‐CT is clearly not indicated for the diagnosis or staging of clinical stage I‐III CC nor for the routine surveillance of these patients following curative surgery, regardless of their risk for recurrence .^1^, ^3^, ^6^

Patients with pathological stage III CC, however, may represent a unique high‐risk group, in which early postoperative PET‐CT may be able to detect metastatic disease that would have been otherwise missed by routine staging, and improve treatment selection for the entire group. Indeed, an earlier study from our group demonstrated that early postoperative PET‐CT modified the staging and treatment of 15% of patients with pathological stage III CC and high‐risk factors for systemic spread.^7^ The sensitivity and specificity of PET‐CT for detecting metastatic disease in these patients were 100% and 69%, respectively.^7^

Following this study, we modified our routine practice to perform, when possible, routine postoperative PET‐CT in all patients with pathological stage III CC, regardless of their disease characteristics, prior to initiation of adjuvant treatment. The aim of the current study was to summarize our experience and thereby to assess the potential benefit of routine early postoperative PET‐CT for the general population with pathological stage III CC.

## 2 MATERIALS AND METHODS

### 2.1 Patients

The registry of the Davidoff Cancer Center (DCC) at Rabin Medical Center (RMC) was screened for all patients who underwent a curative (R0) resection with histologically confirmed pathological stage III CC between 2005 and 2017. Staging was done according to the 7th edition of the American Joint Committee on Cancer (AJCC) staging system. Patients were considered eligible if they had preoperative chest and abdominopelvic CT scans with no evidence of distant metastases, no preoperative PET‐CT, normal postoperative tumor markers and postoperative PET‐CT done within 4 months from surgery, and always before adjuvant chemotherapy, if given, was initiated. Patients with tumors located in the upper third of the rectum were eligible as long as these did not require radiotherapy. While it is our institute's routine policy for over a decade that all pathological stage III CC patients shall undergo an early PET‐CT evaluation before adjuvant treatment, this is not always achieved. As the test is currently not recommended by international guidelines for this indication, the main reasons for not performing PET‐CT in this setting are reimbursement issues, long interval from surgery and the need to avoid further delay in adjuvant treatment and patient non‐compliance. Patients' clinicopathological characteristics, PET‐CT findings, treatment details, and survival outcomes were collected from DCC and RMC electronic databases and from patients' medical records. The study was approved by the institutional ethics committee prior to any research procedures.

### 2.2 PET‐CT protocol

All patients underwent FDG‐PET‐CT on a GE Discovery STE Whole Body PET‐CT scanner (GE Medical Systems, Milwaukee, WI, USA) after 4 hours of fasting with the exception of liberal water intake. Blood glucose levels were examined before ^18^F‐FDG injection to ensure a blood glucose level <200 mg/dL. CT images were acquired at 120 kV and 80 mA, pitch 1.75, 0.8 seconds per tube rotation, and slice thickness of 3.75 mm. During whole‐body CT examination, 80 mL of contrast agent (Ultravist 300, Schering AG, Berlin, Germany) was administered intravenously to ensure fully diagnostic CT data. The PET scan was performed 50‐70 minutes after intravenous injection of 370 to 666 MBq (10‐18 mCi) according to patient's weight. The contrast‐enhanced CT was used for attenuation correction of the PET data. PET was performed from head to mid‐thigh, 2‐3 min per bed position, resulting in a total PET scan time of approximately 20‐25 min (seven or eight bed positions). PET images were reconstructed using an iterative algorithm, with CT‐based attenuation correction applied.

### 2.3 PET‐CT results and work‐up

A positive result was defined as abnormal FDG uptake in the postoperative PET‐CT scan. True positive scans were distinguished from false positive ones on the basis of further investigation (imaging, tissue biopsy) or patients' follow‐up (findings proven over time to truly represent metastatic lesions). Findings interpreted as clear postoperative changes did not require any further assessment but were defined as such by follow‐up imaging in all cases. Patients were labeled “True stage III” if the postoperative PET‐CT did not result in upstaging or “True stage IV” in case the scan revealed metastatic disease.

### 2.4 Statistical analysis

Data were analyzed using the Statistical Package for the Social Sciences 22.0 (SPSS^®^), at a significance level of 0.05. Chi‐square test or Fisher's exact tests were used for categorical data, and the Mann‐Whitney *U* test or Student's *t* test was used for continuous data. Survival was estimated using the Kaplan‐Meier method.

## 3 RESULTS

### 3.1 Patients

**Figure 1:**
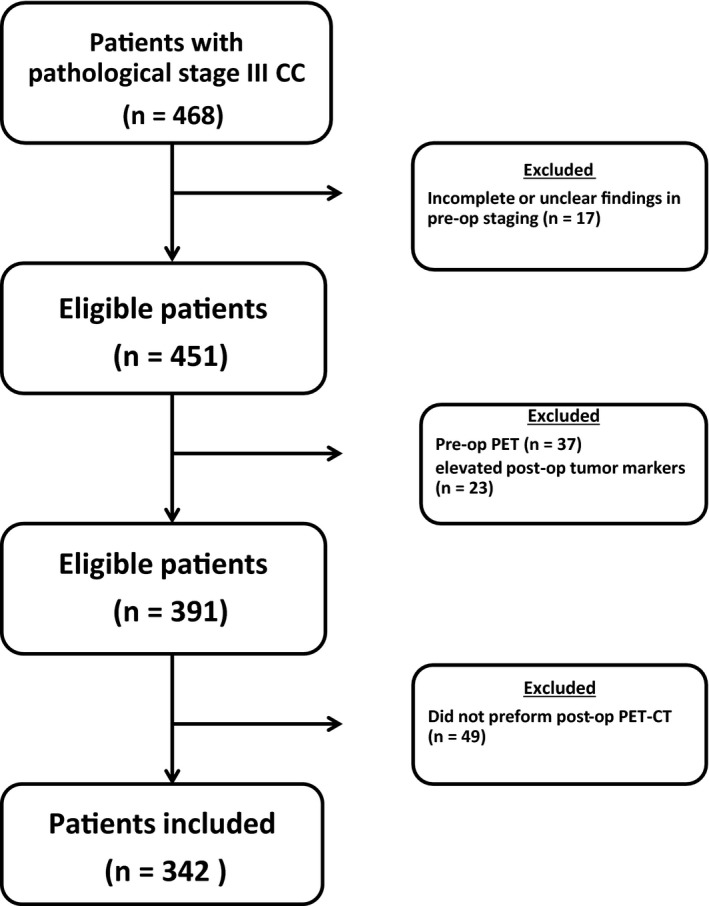
Patients flow

During the study period, 468 patients with pathological stage III CC were treated at DCC. Of those, 77 patients who had inadequate or inconclusive preoperative staging, a preoperative PET‐CT or elevated postoperative tumor markers, were considered ineligible for routine early postoperative PET‐CT and were excluded. Three‐hundred and ninety‐one patients (86.7%) met the study eligibility criteria, of whom 342 (87.5%) actually performed the test and constituted the study population (Figure 1). Patient clinicopathological characteristics are shown in Table 1. One hundred and sixty‐six patients (48.5%) were males and 176 were females (51.5%), and the median age was 66 years (range, 29‐90). The majority of tumors was located in the left colon and upper rectum (63.4%) and was well or moderately differentiated (78.1%). Most patients presented with an advanced T stage (93.2% T3‐T4) and earlier N stage (64.6% N1). Eight patients (2.3%) had N1C disease. The median number of lymph nodes retrieved was 14 (range, 3‐54) and that of positive lymph nodes was 2 (range, 0‐32). Most patients (94.7%) had stage IIIB‐IIIC disease. Preoperative CEA and CA‐19.9 levels, available in 58.8% and 32.7% of cases, respectively, were elevated in 29.8% and 6.6%, respectively.

**Table 1:** Clinicopathological characteristics, n = 342

Age (median, range)	66 (29‐90)
Male gender (n, %)	166 (48.5%)
Tumor location (n, %)	
Right colon	114 (33.3%)
Transverse colon	11 (3.2%)
Left colon/upper rectum	217 (63.4%)
Histology (n, %)	
Adenocarcinoma NOS	271 (79.2%)
Mucinous	58 (17.0%)
Signet ring cell	13 (3.8%)
Grade (n, %)	
1	26 (8.1%)
2	224 (70.0%)
3	68 (21.2%)
4 (anaplastic)	2 (0.6%)
T Stage (n, %)	
1	4 (1.2%)
2	19 (5.6%)
3	311 (90.9%)
4	8 (2.3%)
N Stage (n, %)	
1A	94 (27.5%)
1B	119 (34.8%)
1C	8 (2.3%)
2A	68 (19.8%)
2B	53 (15.5%)
LN examined (median, range)	14 (3‐54)
Positive LN (median, range)	2 (0‐32)
TNM pathological stage (n, %)	
IIIA	18 (5.3%)
IIIB	257 (75.1%)
IIIC	67 (19.6.%)
LVI/VVI (n, %)	72 (21.7%)
PNI (n, %)	41 (12.3%)
ECE (n, %)	69 (20.8%)
Perforation (n, %)	15 (4.7%)
Pre‐op CEA (median, range)	2.8 (0‐163)
Pre‐op CA19‐9 (median, range)	10.3 (0.1‐344.6)

CEA, carcinoembryonic antigen; CA19‐9, cancer antigen 19‐9; ECE, extracapsular extension; LN, lymph node; LVI, lymphovascular invasion; NOS, not otherwise specified; PNI, perineural invasion; VVI, venovascular invasion.

Data were missing on grade (22 patients), PNI (10), ECE (11), LVI/VVI (11), pre‐op CEA (141), and pre‐op CA19‐9 (230).

### 3.2 PET‐CT results and impact

The median time from surgery to PET‐CT scan was 1.5 months (range 0.3‐3.7 months); in 83.9%, the scan was done within 2 months from surgery. The results of the scans are summarized in Table 2. High FDG uptake was observed in 81 patients (23.7%). Twenty patients (5.8%) had clear postoperative changes and required no further work‐up. Thirteen (3.8%) had a false positive abnormal uptake, including high FDG uptake in the tumor bed, non‐regional lymph nodes, and focal lung opacities. Ten patients’ scans were considered to be of low suspicion for malignancy and were closely monitored by further imaging studies and tumor markers and 3 underwent invasive diagnostic procedures to exclude metastatic disease. None of these patients had work‐up related complications. Two patients had abnormal uptake due to pelvic infections. Postoperative PET‐CT modified the management of 46 patients (13.4%) who were found to have true positive findings. Second primary tumors were discovered in 9 patients (2.6%), including two papillary thyroid cancers, a thymoma, an esophageal adenocarcinoma, a small bowel carcinoid, and 4 cases of additional colonic tumors/dysplastic polyps despite a preoperative colonoscopy. Thirty‐seven patients (10.8%) were found to have overt metastatic disease, usually in the liver (48.6%), distant lymph nodes (24.3%), peritoneum (18.9%), or lungs (16.2%). Of the 37 patients found to have metastatic disease, 14 (37.8%) were treated with curative intent, including resection of hepatic (78.6%), pelvic (14.2%), and pulmonary (7.1%) metastases. The 23 metastatic patients treated with palliative intent were found to have metastases in multiple sites (34.8%), non‐regional lymph nodes (21.7%), peritoneum (21.7%), lungs (17.4%), and liver (4.3%).

**Table 2:** PET‐CT scan results and patient outcome, n = 342

PET‐CT results	N (%)
Normal Scan	261 (76.3%)
Abnormal Scan, High FDG Uptake	81 (23.7%)
Postoperative changes	20 (5.8%)
False positive findings	13 (3.8%)
Pelvic Infections	2 (0.6%)
True positive findings	46 (13.4%)
2nd primary	9 (2.6%)
Metastatic	37 (10.8%)
Metastatic, curative intent	14 (37.8%)
Metastatic, palliative intent	23 (62.2%)
Patient outcome	
All patients	342 (100%)
Median OS	Not reached
5‐y OS	77.8%
6‐y OS	77.1%
3‐y DFS	77.8%
5‐y DFS	77.6%
True Stage III	305 (89.2%)
Median OS	Not reached
5‐y OS	81.8%
6‐y OS	81.0%
3‐y DFS	83.1%
5‐y DFS	81.0%
True Stage IV	37 (10.8%)
Median OS	57.2 mo
5‐y OS	47.2%

FDG, Fluorodeoxyglucose; OS, overall survival.

### 3.3 Patient outcome

**Figure 2:**
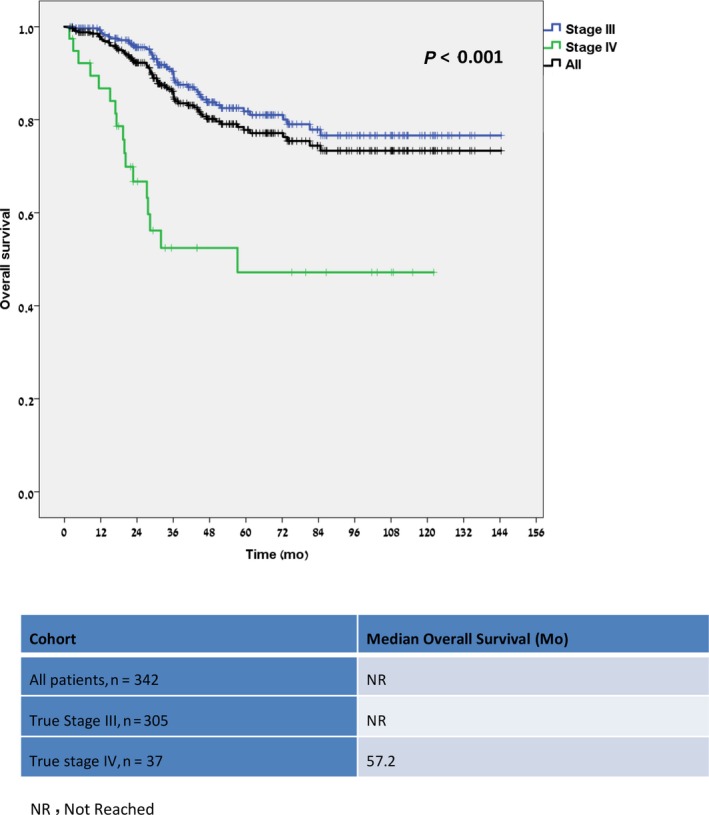
Overall survival by postoperative PET‐CT Staging (n = 342)

**Figure 3:**
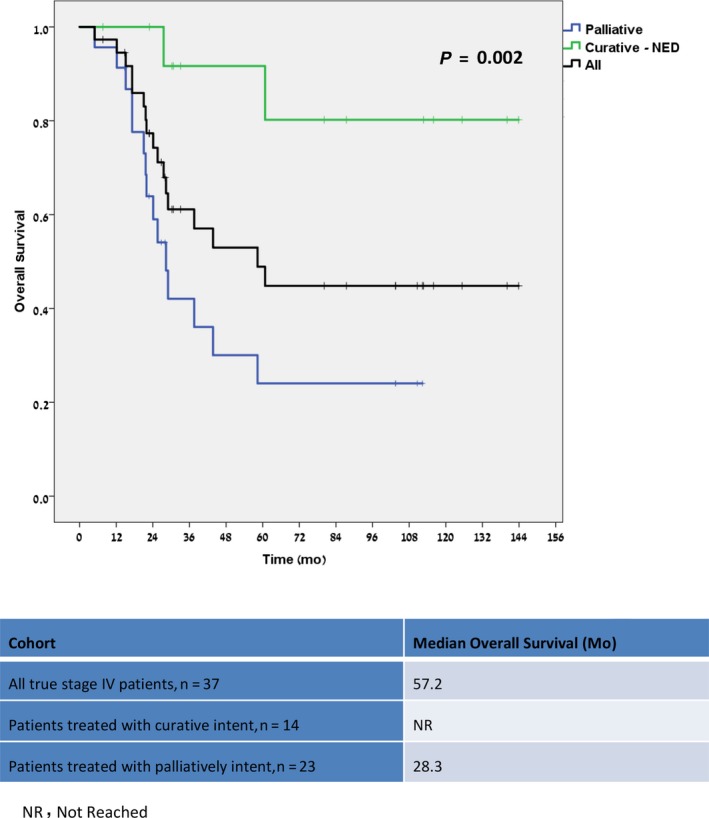
Overall survival for true Stage IV patients by treatment aim (n = 37)

The median follow‐up for the entire study cohort was 48.9 months (range, 1.6‐144.4). As seen in Table 2, patient outcome was analyzed in the different groups, that is, the entire study population, patients with true stage III and stage IV, and patients with true stage IV undergoing potentially curative surgeries. Median overall survival (OS) was similar in the entire cohort and true stage III patients (median not reached for both), and both groups had a significant better OS than patients who were found to have metastatic disease (57.2 months, *P* < 0.0001) (Figure 2). The estimated 5‐year disease‐free survival (DFS) and 6‐year OS rates were 77.6% and 77.1% for the entire cohort and were both 81% for the true stage III patients. Of the true stage IV patients treated with curative intent, 9/14 (64.3%) remain with no evidence of disease (NED), with a median follow‐up of 83.8 months. The 3‐year progression‐free survival (PFS) rate for those patients was 58.6%, and their median OS has not been reached (Figure 3). The estimated 5‐year OS rates of patients with true stage IV was 47.2%, with a significant difference between patients undergoing curative treatment and those who did not (91.7% vs 24.0%, *P* = 0.002) (Figure 3).

### 3.4 Predictive factors

Multiple patient and tumor characteristics, some with well‐established prognostic impact in CC, were evaluated for their ability to predict true positive postoperative PET‐CT. Several factors were indeed associated with a significantly higher chance for upstaging following PET‐CT (Table 3). These included high tumor grade (*P* = 0.009), N2 (*P* = 0.027), stage of disease (*P* = 0.022), presence of perineural invasion (PNI, *P* = 0.007), lymph or venous vascular invasion (LVI/VVI, 0.006), extracapsular extension (ECE, *P* = 0.041), and tumor perforation (*P* = 0.011). Of note, some of these factors were related with very high risk, of more than 20% in some, for true stage IV disease. For example, patients with tumor perforation had a 31.3% risk of positive PET‐CT scans, compared with only 10.4% of those with unperforated tumors (*P* = 0.011).

**Table 3:** Predictive factors for true stage IV, n = 342

Factor	N	Stage 4 (%)	*P*‐value
Gender			
Male	166	10.8	0.752
Female	176	11.9	
T stage			
T1‐2	23	4.3	0.27
T3‐T4	319	11.9	
N stage			
N1	221	8.6	0.027
N2	121	16.5	
ECE			
No	262	9.9	0.041
Yes	69	18.8	
Stage			
IIIA	18	5.5	0.022
IIIB	257	9.7	
IIIC	67	19.4	
Grade			
1‐2	250	8.8	0.009
3‐4	70	20.0	
PNI			
No	291	10.0	0.007
Yes	41	24.4	
LVI/VVI			
No	266	9.4	0.006
Yes	65	21.5	
Histology			
Non‐signet	330	11.5	0.733
Signet	12	8.3	
Endoscopic obstruction			
No	300	11.3	0.913
Yes	42	11.9	
Perforation			
No	326	10.4	0.011
Yes	16	31.3	
Elevated pre‐op CEA			
No	141	10.6	0.861
Yes	61	11.5	
Elevated pre‐op CA19‐9			
No	96	14.6	0.114
Yes	15	0	

## 4 DISCUSSION

This study evaluated the role of PET‐CT in an as of yet unrecognized clinical indication: routine early postoperative assessment in patients with histopathologically proven stage III CC and no clinical indication of any sort for the test. We found that PET‐CT was indeed able to detect previously unidentified clinically meaningful findings in 13.4% of these patients, including 10.8% with overt metastatic disease and 2.6% with a second primary, and thus to modify their management. More importantly, our study suggests that the use of PET‐CT in this setting may have impacted patients' outcome, with 6‐year OS of 77.1% and 81% for the whole study population and for the true stage III patients, respectively, and 5‐year OS of 47.2% for the true stage IV patients. Furthermore, early PET‐CT was able to identify a small group of patients (n = 14) with resectable disease, of whom two thirds remained free of recurrence after a median follow‐up approaching seven years following accurate staging and treatment. Finally, we were able to identify high tumor grade, N2, stage of disease, PNI, LVI/VVI, ECE, and tumor perforation as factors that correlate with positive PET‐CT findings.

The present study was based on the results of an earlier one from our group, in which early postoperative PET‐CT was found to alter the staging and management in 15% of patients with high‐risk stage III CC.^7^ High‐risk features in that study included the presence of pathological T4 tumors, LVI or PNI, grade 3 or 4, fewer than 12 lymph nodes examined or more than 3 lymph nodes involved and an elevated level of CEA or CA‐19.9 pre or postoperatively .^7^ This striking finding, never reported before, urged us to modify our practice and to recommend early postoperative PET‐CT for the general population of stage III CC patients, the focus of this study. Of note, not all patients in the earlier trial were eligible for the current one; patients with elevated postoperative tumor markers, for whom PET‐CT was clinically indicated, were now excluded, emphasizing the routine use of PET‐CT in this study.

As described, all pathological stage III CC patients treated at our institution are referred routinely for an early PET‐CT evaluation before adjuvant treatment. A major concern with the addition of sensitive imaging studies is the discovery of unclear findings that may result in patient anxiety and unnecessary diagnostic procedures, including invasive ones. However, of the 81 patients (23.7%) of the entire cohort who had high FDG uptake, this was determined as clear postoperative changes in 20 (24.7%) and required no further work‐up, and of the 13 patients (16%) who were eventually considered to have false positive scans, only 3 underwent invasive diagnostic procedures. In summary, only 3/342 patients (0.9% of the entire cohort) had “unnecessary” work‐up as a result of the routine early postoperative PET‐CT.

Regardless of its accuracy measures, any novel imaging strategy is required to demonstrate a significant impact on the patient's clinical course. Lacking an intrinsic control arm in our study, we compared our patients' outcome to well‐established stage III CC references in the literature. With the limitation of cross‐trial comparison, the DFS and OS parameters of the entire cohort in our study seemed to be at least comparable to those reported for the FOLFOX arms in the MOSAIC, AVANT, NSABP C‐08, PETACC‐08, and NCCTG N0147 trials. For example, the 3‐year DFS rate in our study was 77.8%, compared with 72.2%, 76%, 75.1%, 78%, and 74.6% rates in the reference trials.^2^, ^8^, ^9^ Not surprisingly, the 5‐year DFS and 6‐year OS rates of the true stage III patients in our study, 81% for both, were better than those of the entire cohort (77.6% and 77.1%), and were higher than in the reference trial.^12^ Nonetheless, even if the outcome measures of the entire cohort in the current study were only equal to those of the reference trials, this would not have excluded a critical benefit for a specific subgroup (true stage IV patients).

Whether early detection of metastatic disease leads to better survival outcomes is subject to much debate. In our study, 37 patients (10.8% of the entire cohort) were found to have metastatic disease and 14 of them were treated with curative intent. Similarly to patients with true stage III CC, we also compared the outcome of our true stage IV patients to up‐to‐date references in the literature. Indeed, the median OS of these patients was also very encouraging: 57.2 months, compared with 29.9 months in the CALGB 80405 trial 13 and 25.0‐33.1 months in the FIRE‐3 trial.^14^ Lastly, while ours is a small cohort, the 3‐year PFS of the true stage IV patients undergoing potentially curative surgery in our study was 59%, much higher than the 35.4% in the investigational arm of the EORTC 40983 trial.^15^ While encouraging, all of these findings require prospective validation.

If confirmed, the results of the present study suggest a new indication for PET‐CT in CC. Thus far, PET‐CT has been studied in various clinical settings in this disease, including some in which the test was shown to improve patient outcome and became routine. Standard indications for the use of PET‐CT in CC are postoperative evaluation of rising CEA levels^4^, ^5^ and preoperative assessment of potentially resectable liver metastases.^3^ Contrary to these, PET‐CT failed to show an improved patient outcome when used for routine surveillance following resection of stage III or IV disease, despite evidence for earlier detection of the recurrence and increased rate of curative metastasectomies.^1^, ^3^, ^16^ In a systematic review, the authors concluded that the routine use of PET or PET‐CT is not indicated for the diagnosis or staging of clinical stage I‐III colorectal cancer nor for routine surveillance in patients deemed to be at high risk for recurrence following curative surgery.^16^

While it seems that the general population of pathological stage III CC may benefit from an early postoperative PET‐CT, predictive factors for a true positive PET‐CT could better define the target population for this approach, improve its accuracy, and reduce its costs, including health and financial implications of unnecessary medical interventions and patient anxiety. In this study, high tumor grade, N2, stage, PNI, LVI/VVI, ECE, and perforation, among the most prominent adverse histological prognostic features in stage III CC,^1^, ^17^, ^18^ were predictive of true stage IV. As depicted in Table 3, high tumor grade, PNI, LVI/VVI, and perforation carried an especially high risk for metastatic disease, of at least 20%. If validated, it may well be that the long‐recognized prognostic impact of these characteristics largely derives from their correlation with pre‐existing metastases that are missed by standard CT but not by PET‐CT.

The main limitations of this study relate to its retrospective design and lack of a control arm, which are generally associated with methodological biases and difficulties in results interpretation. The most concerning bias in our study is clearly associated with patient selection. As routine, early postoperative PET‐CT is not yet recommended in stage III CC and the test is thus not always reimbursed, and as its performance leads to some delay in commencement of adjuvant treatment, PET‐CT was not done in all of the cases constituting its target population. However, despite these factors, PET‐CT was still performed in a high proportion (87.5%) of the eligible patients, reducing the risk of a significant selection bias. Furthermore, the largely consecutive enrollment of patients into the study, its relatively large size, and the availability of well‐established references in the literature clearly help in the interpretation of its results. The study also has some clear strengths. The large sample size and the overly consecutive patient enrollment are obvious ones. In addition, the reality in which all the patients were treated in a single institution and by a limited number of medical staff increases its homogeneity, especially with regard to patient work‐up and treatment. Lastly, this allowed a long and reliable patient follow‐up (over 4 years), which then enabled good interpretation of PET‐CT findings and evaluation of patients' long‐term outcome.

In summary, this study shows, for the first time, the potential of early postoperative PET‐CT to improve the staging and management of the general population of patients with pathological stage III CC. The test allowed early detection of a 2nd primary tumor and of metastatic disease, including otherwise missed curable lesions, in 13.4% of patients with an unremarkable preoperative CT. Several predictive factors for upstaging following PET‐CT were identified, which may guide future patient selection for postoperative PET‐CT.

Moreover, the results of our study suggest that PET‐CT may have a significant impact on patients' long‐term outcome. If confirmed, these results may indeed become practice changing in stage III CC patients. For that purpose, we are currently conducting a prospective validation study; preliminary results are encouraging, and recruitment is ongoing.

## CONFLICT OF INTEREST

The authors declare no conflict of interest.
